# TLR-4 and CD14 Genotypes and Soluble CD14: Could They Predispose to Coronary Atherosclerosis?

**DOI:** 10.3390/jcdd3010009

**Published:** 2016-03-10

**Authors:** Maria Kalliopi Konstantinidou, Nikos Goutas, Dimitrios Vlachodimitropoulos, Antigoni Chaidaroglou, Demetrios Stefanou, Nikoleta Poumpouridou, Renata Mastorakou, Maria Gazouli, Dimitrios Kyparissopoulos, Chara Spiliopoulou

**Affiliations:** 1 Department of Cardiothoracic Surgery, Royal Brompton and Harefield Trust, 156 Montreal House, Surrey Quays Road, London SE16 7AQ, UK; dstefanou@yahoo.com (D.S.); dimkypar@hotmail.com (D.K.); 2 Department of Forensic Medicine and Toxicology, Athens Medical School, Athens 115 27, Greece; goutasnikos@hotmail.com (N.G.); dvlacho@med.uoa.gr (D.V.); poumpouridounikoleta@gmail.com (N.P.); chspiliop@med.uoa.gr (C.S.); 3 Department of Immunology, Onassis Cardiac Surgery Center, Athens 176 74, Greece; achaidaroglou@yahoo.com; 4 Department of Radiology, Onassis Cardiac Surgery Center, Athens 176 74, Greece; irenmast@yahoo.gr; 5 Department of Biology, Athens Medical School, Athens115 27, Greece; mgazouli@med.uoa.gr

**Keywords:** coronary atheroscerosis, coronary artery disease, Toll-Like-Receptor 4 (TLR-4), CD14, sCD14, PCR-RFLP, ELISA

## Abstract

Background: Inflammatory mechanisms are key to the pathogenesis of atherosclerosis. Functional polymorphisms of TLR-4, Asp299Gly and Thr399Ile, CD14 promoter area C260T polymorphism and plasma levels of soluble CD14 are studied in subjects with Coronary Artery Disease (CAD). Methods: DNA was obtained from 100 human paraffin-embedded aortic specimens, from cadavers with known coronary atheromatosis (Group A) and 100 blood samples from patients with CAD, as detected by cardiac Multi-Detector-row-Computed-Tomography (MDCT) (Group B). Our control group consisted of 100 healthy individuals (Group C). Genotyping was performed by Restriction Fragment Length Polymorphism-Polymerase Chain Reaction (RFLP-PCR). Plasma levels of sCD14 were measured with ELISA. Results: For TLR-4 Asp299Gly and Thr399Ile polymorphisms, no statistically significant differences were observed. Regarding the C260T polymorphism, frequencies of T allele were significantly higher in the control group compared to the case group (*p* = 0.05). The Odds Ratio (OR) showed statistically significant association of TT genotype with healthy individuals (OR 0.25, 95% Confidence Interval CI 0.10–0.62, *p* = 0.0017). Plasma levels of sCD14 in patients with CAD (mean value = 1.35 μg/mL) were reduced when compared to reference value. Conclusions: The studied polymorphisms ofTLR-4 showed no association with CAD. Conversely, the functional polymorphism of CD14 has a statistically significant difference in expression between healthy and affected by CAD individuals.

## 1. Introduction

Atherosclerosis, a partially heritable disorder [1], is a chronic inflammatory disease of the blood vessels and underlies the vast majority of cardiovascular disease. Although the genes involved and their possible associations with risk factors for atherosclerosis are still unclear, it is widely accepted that inflammation and infection play a key role in atherosclerosis [2]. Between 1995 and 2005, death rates from cardiovascular disease declined by 26.4%. However, Coronary Artery Disease (CAD) is the leading cause of death [3] and disability worldwide [4]. Cardiovascular disease still causes more global death and disability than any other pathology, except for infection [5].

Immune and inflammatory mechanisms are believed to be key players in the pathogenesis of atherosclerosis [6]. It has been suggested that chronic infection by Gram-negative microorganisms may contribute to the inflammatory component of atherosclerosis [7]. Endotoxin or lipopolysaccharides (LPS) exerts proatherogenic effects by contributing to low density lipoprotein (LDL) oxidation, foam cell formation and atherothrombosis [8]. Toll-Like-Receptors are members of the interleukin-1 receptor (IL-1R) family [9], an evolutionarily conserved signaling system that is a critical determinant of the innate immune and inflammatory responses against invading pathogens. TLR-4 is one of the major receptors for LPS and is also expressed on B and T cells, which mediate the more complex adaptive immunity via a large repertoire of antigen-specific immunoglobulin-class receptors. Its gene is located in the long arm of chromosome 9 (9q33.1) and consists of four exons. The encoded protein plays a fundamental role in pathogen recognition and activation of innate immunity. Studies using TLR-4 knockout mice have revealed that this receptor mediates cellular responses to cell-wall components of Gram-negative bacteria [10,11]. Additionally, mutations in this gene have been associated with differences in LPS responsiveness. Multiple transcript variants encoding different isoforms have also been found for this gene. TLR4 allows LPS recognition and requires CD14 and MD2 as co-receptors. TLR-4 Asp299Gly and Thr399Ile, two cosegregating missense mutations in the extracellular domain of the receptor of the human TLR-4 gene, and CD14 C260T functional polymorphisms are believed to modulate the activity of this complex.TLR-4 Asp299Gly mutation was shown to disrupt TLR-mediated LPS signaling *in vitro* more severely than TLR4 Thr399Ile mutation [12].

CD14 is a glycosylphosphatidylinositol (GPI)-anchored receptor known to serve as a co-receptor for several TLRs both at the cell surface and in the endosomal compartment. Most of the information available concerns CD14’s role as a co-receptor working along with TLR-4 and facilitating cellular responses to low doses of LPS [13]. CD14 is capable of binding LPS at picomolar concentrations and presenting and transferring it to the TLR-4-MD2 complex for the initiation of the transduction pathway through NF-kB and subsequent internalization of the whole complex [14,15]. CD14 also acts in TLR-4-independent fashion and activates the NFAT pathway with unclear consequences [16]. The CD14 receptor is a 356 amino acid membrane glycoprotein whose C-terminal leader sequence of 28–30 amino acids is replaced by a GPI anchor after translation [17]. Thus, CD14 is not a transmembrane protein but it is anchored to the cellular membrane through the GPI linkage. The membrane-expressed CD14 (mCD14) is present on the surface of mature myeloid cells and differentiation of monocytes into macrophages within different tissues is accompanied by a change in mCD14 receptor number [18,19].

CD14 also exists as a soluble molecule (sCD14) that can be detected with two different molecular weights in serum. Various stimuli induce shedding of the GPI-anchored mCD14, probably mediated by serine proteases such as leucocyte elastase, resulting in sCD14 with a molecular mass of 48–49 kDa. Some CD14 molecules stored intracellularly escape GPI anchor attachment and keep the C-terminal leader sequence resulting in sCD14 with a slightly higher molecular weight of 55–56 kDa [20]. The sCD14s have an important role in LPS mediated activation of CD14 negative cells (e.g., epithelial and smooth muscle cells) but the biological differences between these two soluble forms are unknown [19]. A CD14 co-receptor is needed to activate the intracellular signaling pathways because of the lack of a cytoplasmic domain and the inability of GPI to activate signaling pathways directly [21]. The most important of the CD14 co-receptors is TLR-4.

The CD14 gene promoter contains a biallelic single nucleotide polymorphism (SNP) characterized by the presence of either a cytosine (C) or a thymine (T) at position −159 relative to a major transcription initiation site [22,23]. *In vitro* experiments suggested that this position is nearby a nuclear protein-binding DNA element involved in promoter activation [24]. CD14 C260T functional polymorphism enhances the transcriptional activity and results in a higher CD14 receptor density [13,25,26].

Recently, the functional polymorphisms identified in both CD14 promoter and TLR4 genes have been associated with acute coronary events [12,22,25,27,28]. Thus, atherosclerosis may depend on the intensity of the genetically-determined inflammatory response against pathogens or their antigens. We selected patient groups whose severity of coronary atheromatosis was confirmed either by a post mortem report or by a cardiac MDCT imaging. In order to evaluate whether pattern recognition receptors (PRRs) such as TLR-4 and CD14 contribute to atherosclerosis formation, we genotyped both study and control groups for the Asp299Gly and Thr399Ile polymorphisms of the TLR-4 gene and the C260T CD14 gene polymorphism. Moreover, the concentration of soluble CD14 in serum was measured with an Enzyme-Linked Immunoabsorbent Assay (ELISA) technique in our patients.

## 2. Methods

### 2.1. Patients—DNA Extraction

We initially had access to 100 specimens of aorta, obtained from cadavers with coronary atherosclerosis diagnosed as cause of death on autopsy (Group A). Subsequently we created Group B consisting of 100 adults who had moderate to severe coronary atheromatosis on Multi-Detector-row-Computed-Tomography (MDCT) (Group B). Finally, Group C consisted of 100 healthy individuals.

We genotyped all groups with restriction fragment length polymorphism-polymerase chain reaction (RFLP-PCR) analysis and we also tested Group B for plasma concentrations of sCD14 by means of ELISA.

DNA was obtained from 10 μm sections of 100 paraffin-embedded specimens of cadaveric aorta of Group A subjects, after deparafinization with xylene and ethanol with the NucleoSpin Tissue kit (Macherey Nagel, Düren, Germany). Our specimens were retrieved from the Laboratory of Forensic Medicine and Toxicology, Medical School, University of Athens. Group B patients, who had moderate to severe coronary atherosclerosis as detected on MDCT, had their DNA extracted from EDTA blood samples using the NucleoSpin Tissue kit (Macherey Nagel).

The characteristics of Groups A and B are summarized in Table 1. Cardiovascular risk factors such as hypertension, hyperlipidemia, diabetes, family history of CAD, obesity and smoking habit were retrieved from the clinical history of our patients. Hypertension was defined as a history of systolic blood pressure >140 mm Hg or diastolic pressure >90 mm Hg or use of antihypertensive therapy. Similarly hyperlipidemia was defined as total cholesterol >200 mg/dL or LDL >130 mg/dL, or use of cholesterol-lowering medication. As diabetics, were considered patients with history of diabetes or on antidiabetic regime or insulin or these with confirmed fasting glycaemia >126 mg/dL (7.0 mmol/L). Family history was considered positive if a first degree relative had developed clinical CAD. Smoking was defined as current smoking or a history of >10 pack-years and BMI > 30 kg/m^2^ would suggest that the patient is obese.

We defined severe coronary atheromatosis as luminal stenosis of above 70% in more than one coronary arteries and increased calcium concentration >300—as shown in the MDCT. In addition, patients who had already undergone an intervention, Coronary Artery Bypass Grafting (CABG) surgery or Percutaneous Coronary Intervention (PCI), were considered as having severe coronary atheromatosis. Conversely, luminal stenosis of 50% and up to 70% in one or more coronary arteries, and no previous intervention in the coronary arteries is considered as moderate atheromatosis.

Our control group (Group C) consisted of 100 healthy individuals with no cardiac history whose DNA was isolated from EDTA blood samples (NucleoSpin Tissue kit, Macherey Nagel). All groups were matched for age and gender and all subjects came from the same geographically homogeneous population.

Our study was approved by the ethics committee of the University of Athens and the patients have provided their informed consent to participate. All reported research is conducted in accordance with the principles of Helsinki Declaration.

### 2.2. TLR-4 and CD14 Genotyping

Genotyping in all selected polymorphisms was performed by PCR-RFLP analysis. For the TLR-4 Asp299Gly (rs4986790) and TLR-4 Thr399Ile (rs4986791) polymorphisms, the method reported by Lorenz *et al*. was used [29]. Specific, primers for TLR-4 Asp299Gly were: forward (5′-GATTAGCATACTTAGACTACTACCTCCATG-3′) and reverse (5′-GATCAACTTCTGAAAAAGCATTCCCAC-3′) and for TLR-4 Thr399Ile were forward (5′-GGTTGCTGTTCTCAAAGTGATTTTGGGAGAA-3′) and reverse (5′-CCTGAAGA CTGGAGAGTGAGTTAAATGCT-3′). The underlined bases in both forward primers indicate an altered nucleotide that was introduced in order to create either a *Nco*I (TLR-4 Asp299Gly) or a *Hinf*I (TLR-4 Thr399Ile) (both from New England BioLabs, USA) restriction site, respectively. PCR reactions were run at 95 °C for 5 min followed by 35 cycles at 95 °C for 30 s, 55 °C for 30 s, 72 °C for 30 s, and a final incubation at 72 °C for 5 min. A 15-μL aliquot of the product was digested with the appropriate restriction enzyme and electrophoresed in a 3% agarose gel to identify the TLR-4 alleles on the basis of the respective allele size. After digestion, fragment sizes for carriers of the polymorphic allele decreased from 249 bp (wild-type) to 223 bp for the 299 residue and from 406 bp (wild-type) to 377 bp for the 399 residue.

**Figure 1:**
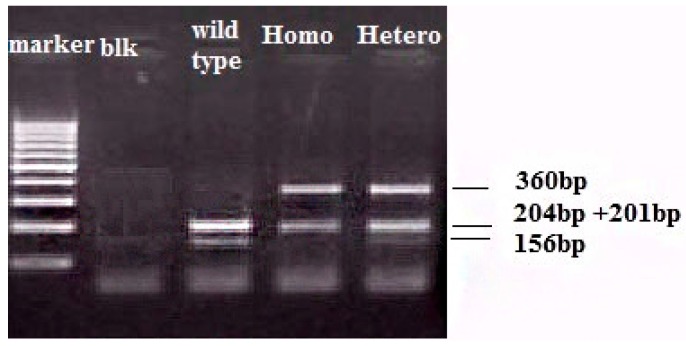
Agarose gel electrophoresis for CD14 C260 T polymorphism genotypes. After digestion of the polymerase chain reaction PCR product with *Hae*III, wild type samples have two bands, at 204 bp, 201 bp (both seen like one more intense band) and 156 bp, homozygotes (Homo) have two bands at 360 bp and 201 bp. Heterozygotes (hetero) should have three visible bands at 360, 204 (+201), 156 bp.

Genotyping for C260T of the CD14 gene (rs2569190) was performed using the method described before [23,30]. In brief, the promoter of the CD14 receptor gene was amplified by the primers CDP-1 (5′-TTGGTGCCAACAGATGAGGTTCAC-3′) and CDP-2 (5′-TTCTTTCCTACACAGCGGCACCC-3′) under the following conditions: an initial denaturation at 95 °C for 5 min, followed by 35 cycles at 92 °C for 40 s, 62 °C for 35 s and 72 °C for 50 s. The final extension step was prolonged to 5 min. The 561 bp PCR product was digested with the restriction enzyme *Hae*III (New England BioLabs, Ipswich, MA, USA), into fragments of 204, 201 and 156 bp in length in the presence of the wild-type allele. The variant allele showed a loss of one *Hae*III cleavage site, resulting in the presence of fragments 360 and 201 bp in length (Figure 1). PCR products were electrophoresed on a 3% agarose gel and visualized by ethidium bromide staining.

### 2.3. ELISA for sCD14

Ethylenediaminetetraacetic acid (EDTA) peripheral blood samples from eighty patients from Group B were drawn. All samples were centrifuged for 10 min in 3000 rpm within 15 min of sampling and plasma was collected and stored at −70 °C until assayed. Soluble CD14 concentrations were determined using a commercially available sCD14 ELISA kit (Quantikine DC140, R&D Systems Inc, Germantown, WI, USA) employing both a monoclonal antibody coated in the microtiter plate and a polyclonal antibody in a sandwich format according to the manufacturer’s instructions. Absorbance values were evaluated with spectrophotometry at 450 nm (ELx800 Universal Microplate Reader, Biotek Instruments Inc, Winooski, VT, USA) using the Lambda KC4 software. Serum levels of sCD14 were expressed as μg/mL. The lower detection limit of the assay was 125 pg/mL and the measurable concentration range was 250 to 16,000 pg/mL. Intra-assay variability was determined by evaluating 3 serum samples 20 times within the same assay run and showed a coefficient of variation (CV) between 4.8% and 6.4%. Inter-assay variability was determined by measuring 3 serum samples in 40 consecutive assay runs and showed a CV between 4.8% and 7.4%.

### 2.4. Statistical Analysis

Allele and genotype frequencies of TLR-4 and CD14 polymorphisms in patients with coronary artery disease and control subjects were calculated. Hardy–Weinberg Equilibrium (HWE) test was performed for each studied SNP and odds ratios (OR) and 95% confidence intervals (CI) for every genotype were calculated for all five inheritance modes (dominant, co-dominant, log-additive, recessive, over-dominant). The analysis was performed with SNP stats online software [31].

Analysis of the association between coronary atherosclerosis and risk factors or the presence of TLR-4 and CD14 polymorphisms was performed with χ^2^ test. The Kolmogorov–Smirnov was used for assessing the distribution of sCD14 data. For comparison of mean values of continuous parametric variables, t test was used. Additionally, the sCD14 values were also divided in two categories depending on whether their copies were above or below the median value and 2 × 2 cross-tabulations were performed (χ^2^ test). All tests were 2-tailed with significance defined at *p* ≤ 0.05. Statistical analysis was performed using the SPSS version 21 (IBM-SPSS Inc., Chicago, IL, USA). Statistical models were assessed by means of the Akaiki Information Criterion (AIC).

## 3. Results

### 3.1. TLR-4 Results

The allele and genotype carrier frequencies for TLR-4-Asp299Gly and TLR-4-Thr399Ile functional polymorphisms are summarized in Table 2 and Table 3.

HWE for the TLR-4-Asp299Gly polymorphism frequency is accepted (*p* > 0.05) while it isnot accepted for the TRL-4-Thr399Ile frequency (*p* = 0.0021). Regarding the TLR-4-Asp299Gly, no statistically significant difference is reached between the frequencies of the polymorphism in Groups A and B (frequency of Gly allele is 0% and 1.50% respectively), compared to the control Group C (frequency of Gly allele is 3%). There is no statistically significant correlation between the presence of the 299Gly allele and severe atheromatosis of the coronary arteries (*p* = 0.1).

For the TRL-4-Thr399Ile polymorphism, there is no statistically significant difference between case and control groups with regards to the frequencies of Ile allele. Allele Ile frequency is only 1% in control group, while the polymorphism does not appear in Groups A or B. Due to the scarcity of the altered allele, more samples should be examined in order to validate any statistically significant conclusions.

### 3.2. CD14—sCD14 Results

Allele and genotype frequencies of the functional polymorphism C260T of the CD14 gene are presented in Table 4. The frequency for functional polymorphism C260T for CD14 shows a HWE value of 0.28, which is accepted. T allele and TT genotype frequencies are significantly higher in the control group compared to the Groups A and B. T allele frequency is 37% for healthy individuals (Group C) while for the case Groups A and B it is 25% and 22.50% respectively −23.75% for the combined Group A + B (*p* = 0.05). The TT (260) genotype exists in 17% of healthy individuals, but only in 5% of patients with coronary atherosclerosis, 3% for Group A and 7% for Group B.

Table 5 shows the CD14 genotype frequencies and statistical results for each inheritance model. The recessive variant has a higher expression in healthy individuals—Group C—with regards to coronary atheromatosis. The Odds Ratio OR, calculated by this model, is 0.25 (95% Confidence Interval CI 0.10–0.62, *p* = 0.0017) and it shows the protective effect for the TT homozygote. Within the case Groups A and B, T allele is detected in slightly higher frequency in the patients with moderate disease compared to patients with severe disease (23% *vs.* 21%, *p* = 0.87).

The mean value of sCD14 levels of Group B patient samples is 1.35 μg/mL, while the median is 1.33 μg/mL (range 1.02–2.10 μg/mL); its distribution in the patient group is normal (Kolmogorov–Smirnov, *p* > 0.5). The reference range for healthy subjects according to the data sheet of the kit is 1.2–2.6 μg/mL with a mean value of 1.8 μg/mL; therefore, plasma levels of sCD14 aresignificantly lower in the examined patients of case Group B than in healthy individuals (*t*-test, *p* < 0.05). Plasma levels of sCD14 are tested in 80 patients from Group B, while they arenot tested in our healthy group and this could be considered as a limitation of our study. The mean plasma concentration of sCD14 in patients with severe disease from Group B islower than in those patients with moderate disease (mean values 1.32 *vs.* 1.40); however, the difference doesnot reach statistical significance (*t*-test, *p* = 0.2). Within the patient Group B, the concentration of sCD14 of TT homozygotes, doesnot differ significantly compared to either the wild-type or to the heterozygotes group (mean sCD14 values in plasma for TT, CT and CC genotypes are 1.37, 1.37, 1.34, respectively). When examining percentages of low or high concentrations of sCD14 in patients with the characteristics of Table 1, no correlation is detected with a x^2^ qualitative test as well as when comparing means of sCD14 with the *t*-test.

## 4. Discussion

### 4.1. TLR-4 Polymorphisms and CAD

TLR-4 may have an important role in the initiation and propagation of atherosclerotic disease [32,33]. In cardiovascular disorders, the role of TLR-4 may be associated with its responsiveness to host ligands, such as heat shock proteins released upon myocardial damage, fibronectin and reactive oxidative species [32,34]. TLR-4 expression has been demonstrated in macrophages and endothelial cells in the atherosclerotic plaque [35].

Several TLR gene polymorphisms have been described. Results from the prospective population-based Bruneck study, showed that patients with the Asp299Gly TLR-4 polymorphism had a significantly lower risk of early plaque development in the carotid arteries [28]. This study also showed that the cumulative burden of cardiovascular disease was reduced by more than half, in patients with both 399Ile and 299Gly TLR-4 polymorphisms, as compared with the background population. Moreover, a decreased risk of acute coronary events has also been reported among carriers of the 299Gly TLR-4 polymorphism [27]. Other studies have reported that this polymorphism imparts protection from carotid and femoral artery atherosclerosis [12,28].

Further indirect support for a protective role of the Asp299Gly TLR4 polymorphism in human atherogenesis derives from the association of this genetic variant with low concentrations of C reactive protein, adhesion molecules, and other acute phase reactant and inflammatory molecules [28].

Analysis of the Southampton Atherosclerosis Study, on the other hand, could not show any association between the TLR-4 Asp299Gly polymorphism and either severity of or susceptibility to coronary artery diseases [36]. Expression of inflammatory markers did not correlate with the Asp299Gly TLR-4 polymorphism. Furthermore, a report from the Stockholm Heart Epidemiology Program found that men with both the Asp299Gly and Thr399Ile polymorphisms had an increased risk of myocardial infarction [37].

A recent study in a Turkish population suggests that both the TLR-4-Asp299Gly and Thr399Ile polymorphisms are not associated with the presence and the severity of CAD [38]. Exploratory analyses investigated associations between the TLR-4 polymorphism and death/non-fatal MI after a mean follow-up of 4.9 years. After adjustment for diabetes and other covariates, no significant effect of 299Gly carriage was observed for either death or death/MI [39]. Same results were obtained in other studies as well [38,40,41].

In our study we could not establish any association between either the Asp299Gly TLR-4 polymorphism or the Thr399Ile polymorphism and the severity of atheromatous disease such as was shown in pathology specimens or radiological examination. The frequency of the Asp299Gly TLR-4 polymorphism does neither differ significantly between the case Groups A and B and the control group nor does it correlate with the severity of the disease or the other risk factors we examined. The Thr399Ile functional polymorphism is not detected in our case group. Due to the scarcity of the altered alleles, more samples should be examined in order to reach statistically significant conclusions.

### 4.2. CD14 Polymorphisms, sCD14 and CAD

There is a growing body of experimental evidence that implicates that a single nucleotide polymorphism in the CD14 promoter might contribute to the polygenic susceptibility towards atherogenesis in humans. The polymorphism C260T of the CD14 promoter has been shown to increase transcriptional activity [25]. This enhanced transcriptional activity has been associated with higher concentrations of sCD14 and enhanced expression of mCD14 on monocytes, and with the risk of MI [22,23].

The CD14 receptor is considered to be a monocyte activation marker and both increased density of mCD14 and serum concentrations of sCD14 have been reported in patients with acute coronary syndromes [42,43]. However, it has been shown that the CD14 receptor is not merely a monocyte activation marker, as it can also synergize with C-reactive protein in the activation of endothelium, considered to be the first step in atherogenesis and coronary events.

Studies investigating the association between the CD14 C260T polymorphism and coronary disease have produced inconsistent results so far.

Unkelbach *et al.* [22] found an increased risk of MI among homozygous carriers of the T allele with a low atherosclerotic risk profile. Other studies involving Czech [23] and Japanese [44] populations showed an increased risk of MI associated with the T allele and the TT genotype of the CD14 promoter polymorphism.

Conversely, the CC genotype was associated with an increased occurrence of incident coronary occlusion and increased carotid artery intima media thickness (IMT) [45,46] Another prospective study did not find any association between the polymorphism and the risk of coronary events [47]. A recent meta-analysis has suggested a possible association between the CD14 C260T polymorphism and ischemic heart disease risk in an East Asian population but not in European and Indian populations [48].

In our study, for the population we studied, we concluded that T allele and the TT genotype frequencies are increased among healthy individuals as compared to patients with atheromatosis of their coronary arteries. T allele frequency is 37% for healthy individuals (Group C) while for the case Groups A and B it is 23.75% (*p* = 0.05). The TT genotype is found in 17% of healthy controls, but only in 5% of patients with coronary atherosclerosis—3% for Group A and 7% for Group B. CD14 C260T promoter polymorphism could be implicated in protective pathways against CAD. Furthermore, if our findings are supported by more prospective studies, this polymorphism may be used as a genetic susceptibility marker of atherosclerosis. Further studies are required to validate these points.

Few data are available on the association between sCD14 and the risk of coronary disease. In a large case–control study, plasma sCD14 levels did not differ between individuals with stable angina pectoris and controls [49]. No relationship was found between plasma concentrations of sCD14 and stable coronary artery disease or carotid IMT [49,50]. Another prospective study (the PRIME Study) did not provide evidence for an association of sCD14 with a future coronary event in a cohort of healthy men [51]. Moreover, individuals with severe coronary events, during follow-up, showed plasma sCD14 concentrations similar to those of cases suffering from angina pectoris.

We examined the sCD14 levels in 80 patients from Group B. The mean value of sCD14 of all Group B patient samples (1.35 μg/mL) islower than the mean reference value reported for healthy individuals by the kit manufacturer (1.8 μg/mL). sCD14 concentrations were not further tested in our control group. The kit manufacturer does not clarify which form of sCD14 is detected by the combination of antibodies, or the overall level of efficiency. The aim of our study was to identify whether the plasma levels of sCD14 were correlated with the severity of atherosclerosis in our patients, as we hypothesized that this could become a possible indicator of severe disease. Among patients with severe and moderate atheromatosis, plasma levels of sCD14 differed only slightly (1.32 *vs.* 1.40, *p* = 0.2). Since the T allele is expected to produce more CD14 mRNA and assumingly more sCD14, this result is consistent with the fact that more T alleles are present in the patient group with moderate rather than severe coronary atheromatosis (23% *vs.* 21%). No association isdetected between sCD14 levels and either genotype or any other patient characteristic, as described in Table 1. We cannot exclude the possibility that other factors than those we examined, might contribute to the reduction in the concentration of sCD14 in our patients, compared to reference values or to the fluctuations in concentration observed between patients with severe or moderate disease. Further studies of larger scale in different populations should be conducted since we now have the first signs that sCD14 could well be a prognostic biomarker.

The advances in DNA testing make the use of functional polymorphisms appealing in detection of genetic predisposition of coronary atheromatosis. Rapid advances in genomic, proteomic, and metabolomic technology provide researchers with valuable tools for understanding the genetic predisposition to atherosclerotic cardiovascular disease [52].

## 5. Conclusions

Cardiovascular disease remains the main cause of death in developed countries. Therefore, the need to further investigate the underlying causes and genetic factors is as imminent as ever. Our study of the functional polymorphisms of TLR-4, Asp299Gly and Thr399Ile, and the polymorphism of the CD14 promoter area, C260T—in order to evaluate their association with the accumulation of coronary atheromatosis—could encourage the expansion of research to larger, different and more diverse populations. Possible biomarkers such as sCD14 that can be easily quantified in plasma, would be of fundamental importance for early treatment or even prevention of clinical manifestations of coronary artery disease in the near future.

## Figures and Tables

**Table 1:** Clinical characteristics of all 200 patients (Patients in Groups A and B).

Clinical Characteristics	N (%)
Mean Age in Years (SD)	49.53 (6.9)
Age Group	
≤50 years	86 (43)
>50 years	114 (57)
Gender	
Men	163 (81.5)
Women	37 (18.5)
Severity of atherosclerosis	
Moderate	89 (44.5)
Severe	111 (55.5)
Smoking	
Yes	50 (25)
No	150 (75)
Family History	
Yes	101 (50.5)
No	99 (49.5)
Dyslipidemia	
Yes	127 (63.5)
No	73 (36.5)
Hypertension	
Yes	94 (47)
No	106 (53)
Diabetes	
Yes	28 (14)
No	172 (86)
BMI	
≤30 kg/m^2^	92 (46)
>30 kg/m^2^	108 (54)

**Table 2:** Frequencies of Toll-Like-Receptor-4/Asp 299 Gly polymorphism.

Polymorphisms of TLR-4										
Group	Results			Frequency			Results		Frequency	
	ASP/ASP	ASP/GLY	GLY/GLY	ASP/ASP	ASP/GLY	GLY/GLY	A ALLELE	G ALLELE	A ALLELE	G ALLELE
**A**	100	0	0	100.0%	0.0%	0.0%	200	0	100.00%	0.00%
**B**	97	3	0	97.0%	3.0%	0.0%	197	3	98.50%	1.50%
**A+B**	197	3	0	98.5%	1.5%	0.0%	397	3	99.25%	0.75%
**C**	95	4	1	95.0%	4.0%	1.0%	194	6	97.00%	3.00%

**Table 3:** Frequencies of Toll-Like-Receptor-4/Thr 399 Ile polymorphism.

Polymorphisms of TLR-4										
Group	Results			Frequency			Results		Frequency	
	THR/THR	THR/ILE	ILE/ILE	THR/THR	THR/ILE	ILE/ILE	T ALLELE	I ALLELE	T ALLELE	I ALLELE
**A**	100	0	0	100.00%	0.00%	0.00%	200	0	100.00%	0.00%
**B**	100	0	0	100.00%	0.00%	0.00%	200	0	100.00%	0.00%
**A + B**	200	0	0	100.00%	0.00%	0.00%	400	0	100.00%	0.00%
**C**	99	0	1	99.00%	0.00%	1.00%	198	2	99.00%	1.00%

**Table 4:** Frequencies of CD14/C-260 T polymorphism.

Polymorphisms of CD14										
Group	Results			Frequency			Results		Frequency	
	CC	CT	TT	CC	CT	TT	C ALLELE	T ALLELE	C ALLELE	T ALLELE
**A**	53	44	3	53.00%	44.00%	3.00%	150	50	75.00%	25.00%
**B**	62	31	7	62.00%	31.00%	7.00%	155	45	77.50%	22.50%
**A + B**	115	75	10	57.50%	37.50%	5.00%	305	95	76.25%	23.75%
**C**	43	40	17	43.00%	40.00%	17.00%	126	74	63.00%	37.00%

**Table 5:** Genotype frequencies, inheritance models and calculated Odds Ratios for CD14 polymorphism C260T.

Model	Genotype	Controls	Patients	OR (95% CI)	*p*-Value	AIC
**Codominant**	C/C	43%	57.9%	1.00	**0.0031**	325.8
	C/T	40%	37.2%	0.69 (0.40–1.20)		
	T/T	17%	4.8%	0.21 (0.08–0.055)		
**Dominant**	C/C	43%	57.9%	1.00	**0.021**	330
	C/C-C/T	57%	42.1%	0.55 (0.33–0.92)		
**Recessive**	C/C-C/T	83%	95.2%	1.00	**0.0017**	325.5
	T/T	17%	4.8%	0.25 (0.10–0.62)		
**Overdominant**	C/C-T/T	60%	62.8%	1.00	0.66	335.1
	C/T	40%	37.2%	0.89 (0.53–1.50)		
**Log-additive**	-	-	-	0.54 (0.36–0.80)	**0.0017**	325.4
